# Cost-effectiveness analysis of second-generation androgen receptor antagonists for the treatment of metastatic hormone-sensitive prostate cancer

**DOI:** 10.3389/fpubh.2025.1680002

**Published:** 2025-10-16

**Authors:** Yang Yang, Ya-Qing Chen, Long-Zhuan Huang, Yong Chen

**Affiliations:** Key Specialty of Clinical Pharmacy, The First Affiliated Hospital of Guangdong Pharmaceutical University, Guangzhou, China

**Keywords:** second-generation AR antagonists, enzalutamide, apalutamide, darolutamide, metastatic prostate cancer, Markov model, cost-utility analysis

## Abstract

**Objective:**

The combination of second-generation androgen receptor (AR) antagonists with androgen deprivation therapy (ADT) has shown good efficacy and safety in advanced prostate cancer. This study aims to evaluate the cost-effectiveness of three second-generation AR antagonists in the treatment of metastatic hormone-sensitive prostate cancer (mHSPC) in China, providing pharmacoeconomic evidence for clinical drug selection.

**Methods:**

A Markov model was constructed based on data from the ARCHES, TITAN, and ARANOTE phase III clinical trials, with a 28-day cycle period. Direct medical costs and quality-adjusted life years (QALYs) were simulated over a 15-year horizon. The incremental cost-effectiveness ratio (ICER) was used as the primary outcome, and a willingness-to-pay (WTP) threshold of three times the 2024 per capita GDP of China was set for cost-utility analysis. Sensitivity analysis was conducted to validate the model’s influencing factors and the robustness of the results.

**Results:**

The cumulative cost of the apalutamide regimen was ¥776,807, resulting in 4.95 QALYs. Compared to apalutamide, the ICER for enzalutamide was ¥643,309/QALY, while for darolutamide, the ICER was -¥40,625/QALY.

**Conclusion:**

For Chinese mHSPC patients, darolutamide is the most cost-effective treatment at a WTP threshold of ¥287,391/QALY, followed by apalutamide, with enzalutamide being less favorable.

## 1 Introduction

The GLOBOCAN 2022 data released by the International Agency for Research on Cancer in 2024 shows that prostate cancer is the second most common malignancy among men worldwide and ranks eighth in mortality among 36 types of cancer. In China, the incidence of prostate cancer in men aged 60 and above has shown a significant upward trend (1), and its diagnosis and treatment costs have placed a substantial economic burden on both patients and the healthcare system (2). Metastatic hormone-sensitive prostate cancer (mHSPC) refers to prostate cancer that has metastasized at diagnosis and has not yet received endocrine treatment. It can be divided into low-volume and high-volume diseases. In China, approximately 54% of patients are diagnosed with distant metastasis, indicating advanced disease (3). Although most patients respond well initially to androgen deprivation therapy (ADT), the majority will still progress to metastatic castration-resistant prostate cancer (mCRPC) within 1–3 years (4).

In recent years, with continuous advancements in medical technology, novel endocrine therapies—particularly second-generation androgen receptor (AR) antagonists—have provided new treatment options for patients with advanced prostate cancer. Second-generation AR antagonists demonstrate a more comprehensive mechanism of action compared to first-generation AR antagonists, which significantly improve patients’ prognosis and delay disease progression, and have been widely recognized and applied globally. Currently, four second-generation AR antagonists are available in China. Enzalutamide, apalutamide, and darolutamide have all received approval from the U.S. Food and Drug Administration (FDA). The fourth drug, rezvilutamide, a domestically developed medication, has been approved by China’s National Medical Products Administration (NMPA) but has not yet obtained FDA clearance. It is noteworthy that rezvilutamide is currently approved only for patients with high-volume mHSPC.

In the treatment of mHSPC, the *CSCO Guidelines 2024* (5) and the *NCCN Guidelines Version 2.2025* (6) have parallel recommendations for three second-generation AR antagonists approved by the FDA. Unlike the treatment regimen of enzalutamide or apalutamide combined with ADT, the standard regimen for darolutamide is a triple combination of ADT and docetaxel, though the toxicity of docetaxel limits its clinical use. The latest ARANOTE study (7) confirms that the darolutamide + ADT combination regimen can improve radiographic progression-free survival (rPFS) in mHSPC patients. A network meta-analysis (8, 9) shows that its efficacy is not significantly different from that of the triple combination regimen, while offering better safety. Real-world studies in China (10) also support its good efficacy and safety. The indication application for darolutamide + ADT in the treatment of mHSPC has been submitted globally. As treatment options increase, conducting pharmacoeconomic evaluations of second-generation AR antagonists is of great significance for assessing the value of the drugs, optimizing treatment options, and alleviating the financial burden on patients and healthcare security systems.

As an important method for improving clinical drug management and optimizing healthcare resource allocation, pharmacoeconomic evaluation methods include cost-effectiveness analysis, cost-utility analysis, cost-benefit analysis, and minimum cost analysis (11). Cost-utility analysis, as a subset of cost-effectiveness analysis (12), uses quality-adjusted life years (QALYs) as the health output indicator, and both methods use incremental cost-effectiveness ratio (ICER) as the evaluation metric, which refers to the additional cost required to gain one additional unit of health output (13). Cost-utility analysis is widely applied in the pharmacoeconomic evaluation of oncology drugs.

Given the differences in efficacy, safety, and cost of the second-generation AR antagonist combined with ADT treatment for mHSPC patients, and the fact that previous studies have only evaluated the economic viability of darolutamide + ADT + docetaxel (14, 15), there has been no economic study on the treatment of mHSPC with darolutamide + ADT. This study conducts a cost-utility analysis of second-generation AR antagonists for the treatment of mHSPC, based on the *Chinese Pharmacoeconomic Evaluation Guidelines 2020* (16), from the perspective of the Chinese healthcare system, incorporating domestic drug pricing and residents’ income levels. The aim is to provide a basis for clinical, rational drug use and healthcare cost control.

## 2 Methods

### 2.1 Patient characteristics

Since there are no head-to-head clinical trials between second-generation AR antagonists, a network meta-analysis is required to further compare survival data. To this end, this study systematically searched the English databases PubMed, Embase, and self-built databases for literature published up to January 2025, as well as relevant conference reports from both domestic and international sources. In clinical trials for mHSPC, the ENZAMET, China AECHES, and ARASENS trials were excluded from the analysis because they did not fully publish rPFS and overall survival (OS) curves (17–21). The clinical trial of rezvilutamide (CHART) only included patients with high tumor burden (22), and the baseline characteristics differed from those of other second-generation AR antagonists’ clinical trials (which included both high and low tumor burdens), so rezvilutamide was also excluded from the cost-utility analysis. Finally, three Phase III randomized controlled trials—ARCHES (23, 24), TITAN (25, 26), and ARANOTE (7)—were included. The Markov model constructed for this study simulated a population whose characteristics were consistent with those of the populations in the aforementioned trials: ① histologically diagnosed as hormone-sensitive prostate cancer with confirmed metastasis through imaging (bone scan/CT/MRI); ② Eastern Cooperative Oncology Group performance status score of 0–1.

### 2.2 Clinical trial treatment regimen

#### 2.2.1 Initial treatment for mHSPC patients entering the model

All patients receive ADT as the base treatment, combined with enzalutamide, apalutamide, or darolutamide.

#### 2.2.2 The treatment after disease progression

Based on the design of each trial, subsequent treatment options include: docetaxel + ADT, abiraterone acetate + ADT, enzalutamide + ADT, apalutamide + ADT.

### 2.3 Model structure

**Figure 1:**
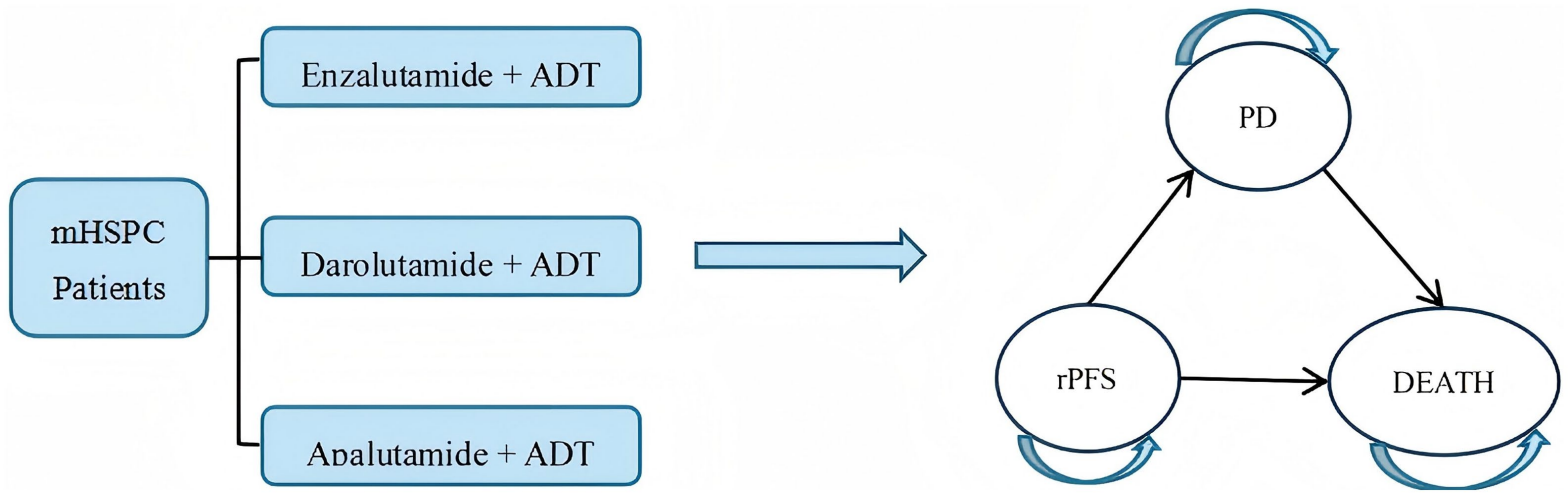
State transition diagram of Markov model.

A Markov model can simulate the long-term, complex progression of diseases flexibly by defining mutually exclusive health states (e.g., disease stability, progression, death) and specifying the transition probabilities between these states. It enables extrapolation of lifetime treatment costs and health utility through cyclical periodic structures. It was widely applied in pharmacoeconomic evaluations of prostate cancer globally (27, 28). Given the multi-state and typically irreversible nature of mHSPC disease progression, this study employed Tree Age Pro 2022 to construct a Markov model incorporating three health states: rPFS, progressive disease (PD), and death. PD was defined as radiological progression: soft tissue lesion progression on CT/MR [RECIST 1.1 (29)] or ≥2 new bone lesions on bone scan [PCWG3 (30)]. To align with clinical reality, disease progression within the model was set as irreversible. The specific state transition rules are as follows: all patients start in the rPFS state; only unidirectional transitions are allowed: rPFS → PD → death or rPFS → death; after transitioning to the PD state, the initial treatment regimen is discontinued, and a preset subsequent treatment regimen is initiated; death is an irreversible endpoint state (see Figure 1).

Based on the ADT administration design used in the included clinical trials, the cycle length of the model was set to 28 days. This study assumes a patient body surface area of 1.72 m^2^ (14), with all patients receiving ADT treatment using leuprolide acetate microspheres for injection. Considering factors such as the median age of clinical trial participants and the average life expectancy in China, the simulation time was limited to 15 years.

### 2.4 Extraction of survival data and calculation of transition probabilities

Data from survival curves published in mHSPC clinical trials were extracted using WebPlotDigitizer software. The rPFS and OS curves for three groups were digitized to obtain the time points and corresponding survival rates for each data point (31), and the data were organized accordingly. After organizing the mHSPC data into a format readable by the “IPDfromKM” package, we used the “IPDfromKM” package in R (v4.4.1) to reconstruct individual patient data, generating Kaplan–Meier survival curves. The survival curves were then fitted and extrapolated using the Weibull distribution to obtain the shape parameter (*γ*) and scale parameter (*λ*) (31, 32).

Due to the lack of head-to-head trials between second-generation AR antagonists, this study adjusted the survival curves for apalutamide and darolutamide based on the enzalutamide group survival curve, using the hazard ratio (HR) values from a network meta-analysis comparing apalutamide and darolutamide (7, 23–26). The survival curves for apalutamide and darolutamide were adjusted by assuming that the *γ* of these curves is equal to that of enzalutamide, and *λ* is equal to the *λ* of enzalutamide multiplied by the HR (other treatment regimens/enzalutamide) (32, 33). The parameter results for the adjusted mHSPC rPFS and OS curves are shown in Table 1.

**Table 1:** Survival curve parameters of mHSPC.

Treatment	rPFS		OS	
	Shape parameter (*γ*)	Scale parameter (*λ*)	Shape parameter (*γ*)	Scale parameter (*λ*)
Enza_ADT	1.131400	0.007900	1.426021	0.001476
Apa_ADT	1.131400	0.006004	1.426021	0.001461
Dar_ADT	1.131400	0.006715	1.426021	0.001830

Transition probability refers to the probability of a patient moving from one state to another during each cycle. Based on whether the transition probability changes over time, it can be classified as static or dynamic. Due to the time-dependent nature of disease progression in cancer patients, the Markov model in this study applied dynamic transition probabilities for analysis. The adjusted *γ* and *λ* parameters were incorporated into the transition probability formula $\mathrm{tp}(t_{u})=1-\exp\{\lambda {\left(t-u\right)}^{\gamma }-\lambda t^{\gamma }\}\;$ (31), yielding time-dependent transition probabilities for different states during each cycle, where *t* represents survival time and *u* represents the cycle period.

### 2.5 Cost and utility value parameters

This study is based on the perspective of the Chinese healthcare system. Due to the difficulty in obtaining direct non-medical costs, only direct medical costs are included, which encompass drug costs, follow-up costs (including laboratory tests and imaging examinations), supportive treatment costs, end-of-life care costs, ≥3 grade adverse events (≥3 AEs) management costs, and subsequent treatment costs (34). Drug costs are sourced from the Guangzhou Drug and Medical Consumables Procurement Platform (https://gpo.gzggzy.cn/ referred to as the “Procurement Platform”). Injection and imaging examination costs are sourced from the Guangzhou Municipal Public Medical Institutions Basic Medical Service Project Price Summary Table (https://m12333.cn/qa/pidzc.html, December 2024, referred to as the “Service Project Price Table”). The price data was retrieved until December 2024, and other cost data come from published literature. The drug dosages, ≥3 AEs incidence rates, and subsequent treatment proportions for the mHSPC treatment regimens are cited from original clinical trial data, as detailed in Table 2.

**Table 2:** Incidence of ≥3 AEs and proportion of subsequent treatment in mHSPC clinical trials.

Project	Enza_ADT	Apa_ADT	Dar_ADT
≥3 AEs incidence rate (%)			
Fatigue	1.7	0	0
Hypertension	3.3	8.4	4.3
Rash	0	6.3	0
Fracture	1	1.3	0
Anemia	0	0	3.1
Back pain	0.9	2.3	1.1
Elevated aspartate aminotransferase (AST)	0	0	2.2
Elevated alanine aminotransferase (ALT)	0	0	2
Neutropenia	0.3	0	0
Proportion of subsequent treatment (%)			
Docetaxel + dexamethasone + ADT	8.4	26.8	22.7
Abiraterone + methylprednisolone + ADT	4.5	14.5	12.8
Enzalutamide + ADT	42	6.5	3
Apalutamide + ADT	0	0	1.5

Health utility values are mostly derived from standardized health-related quality of life measures, such as the EuroQol-5D and SF-6D scales (35, 36). Health utility values are typically represented by numbers ranging from 0 to 1, where 0 represents death and 1 represents perfect health. In addition to the disease itself affecting a patient’s health utility, AEs occurring during treatment can also lower quality of life, thus impacting the health utility value (14). Considering the varying incidence rates of ≥3 AEs among different treatment regimens, the health utility values in this study are adjusted based on the negative utility values caused by ≥3 AEs. Health utility values for different disease states are obtained from published literature related to mHSPC. The parameters for costs, health utility values, and their distributions in the model are provided in Table 3.

**Table 3:** Parameters values of Markov model.

Parameters	Baseline values	Lower limits	Upper limits	Distribution	Sources
Costs of drugs per cycle (yuan)					
Enzalutamide	7795.2	6236.16	9354.24	Gamma	Procurement platform
Apalutamide	5420.8	4336.64	6504.96	Gamma	Procurement platform
Darolutamide	5535.16	4428.128	6642.192	Gamma	Procurement platform
ADT	905.61	724.488	1086.732	Gamma	Procurement platform
Second-line treatment					
Docetaxel + dexamethasone + ADT	3813.51	3050.808	4576.212	Gamma	Procurement platform, service project price list
Abiraterone + methylprednisolone + ADT	1517.2868	1213.829	1820.744	Gamma	Procurement platform, service project price list
Enzalutamide + ADT	8700.81	6960.648	10440.97	Gamma	Procurement platform, service project price list
Apalutamide + ADT	6326.41	5061.128	7591.692	Gamma	Procurement platform, service project price list
Cost of disease management (yuan per visit)					
Follow-up cost_Laboratory tests	782.13	625.704	938.556	Gamma	Reference [38]
Follow-up cost_Imaging tests	394.88	352.54	438.76	Gamma	Service project price list
Supportive treatment	2255.87	1804.696	2707.044	Gamma	Reference [38]
End-of-life care	16306.27	13045.02	19567.52	Gamma	Reference [38]
≥3 AEs management cost (yuan per event)					
Fatigue	540	432	648	Gamma	Reference [39]
Hypertension	114.26	91.408	137.112	Gamma	Reference [40]
Rash	39.1	31.28	46.92	Gamma	Reference [41]
Fracture	21,500	17,200	25,800	Gamma	Reference [42]
Anemia	271.7	217.36	326.04	Gamma	Reference [40]
Back pain	73.6	58.88	88.32	Gamma	Reference [40]
Elevated aspartate aminotransferase (AST)	270.84	216.672	325.008	Gamma	Reference [40]
Elevated alanine aminotransferase (ALT)	270.84	216.672	325.008	Gamma	Reference [42]
Health utility value					
rPFS	0.76	0.68	0.84	Beta	Reference [39]
PD	0.68	0.61	0.75	Beta	Reference [43]
Fatigue	−0.115	−0.092	−0.138	Beta	Reference [44]
Hypertension	−0.044	−0.0352	−0.0528	Beta	Reference [45]
Rash	−0.13	−0.104	−0.156	Beta	Reference [44]
Fracture	−0.15	−0.12	−0.18	Beta	Reference [44]
Anemia	−0.07	−0.056	−0.084	Beta	Reference [46]
Back pain	−0.067	−0.0536	−0.0804	Beta	Reference [47]
Elevated aspartate aminotransferase (AST)	−0.057	−0.0456	−0.0684	Beta	Reference [48]
Elevated alanine aminotransferase (ALT)	−0.057	−0.0456	−0.0684	Beta	Reference [48]
Discount rate (%)	5%	0	8%	Beta	Reference [16]
HR_rPFS_AvE (Apa vs. Enza)	0.76	0.26	2.18	Lognormal	Network meta-analysis
HR_rPFS_DvE (Dar vs. Enza)	0.85	0.29	2.48	Lognormal	Network meta-analysis
HR_OS_AvE (Apa vs. Enza)	0.99	0.55	1.79	Lognormal	Network meta-analysis
HR_OS_DvE (Dar vs. Enza)	1.24	0.65	2.36	Lognormal	Network meta-analysis

### 2.6 Cost-utility analysis

The transition probabilities, costs, health utility values, and other parameters mentioned above were input into the Markov model to conduct a cost-utility analysis for three second-generation AR antagonists. The simulation time horizon is 15 years, and to reduce bias caused by the discrete-time assumption, both costs and QALYs were adjusted with half-cycle correction. Based on the *China Drug Economic Evaluation Guidelines 2020* (16), a 5% discount rate was applied to both treatment costs and QALYs. The model output evaluation indicators include the total cost, QALYs, and ICER for each treatment regimen. In this study, three times the 2024 per capita GDP of China (287,391 yuan/QALY) was used as the willingness-to-pay (WTP) threshold. When the ICER is less than the WTP, the treatment is considered cost-effective.

### 2.7 Sensitivity analyses

To assess the robustness of the model results and the impact of parameter uncertainty on the conclusions, this study performed both univariate sensitivity analysis and probabilistic sensitivity analysis.

#### 2.7.1 One-way sensitivity analysis

For parameters derived from the literature, the 95% confidence interval (95% CI) was used as the upper and lower limits. If the 95% CI was unavailable, a fluctuation range of ±20% around the baseline value was applied. For healthcare service prices (e.g., examination and hospitalization costs), a sensitivity analysis was conducted using the fee standards of primary medical institutions (lower limit) and tertiary medical institutions (upper limit). The results were presented using a tornado diagram, showing the impact of each parameter on the outcome and ranking them.

#### 2.7.2 Probabilistic sensitivity analysis

Probability distributions were assigned to the parameters for second-order Monte Carlo simulations. The model was randomly sampled 10,000 times, and the results were presented using cost-effectiveness scatter plots and cost-effectiveness acceptability curves (34).

## 3 Results

### 3.1 Cost-utility analysis

Under the 15-year simulation time horizon, the results of the cost-utility analysis based on the Markov model showed that darolutamide had the lowest total cost (751,369 yuan) and the highest QALYs (5.58 QALYs); enzalutamide had the highest total cost (872,033 yuan) and relatively high QALYs (5.10 QALYs); apalutamide had a mid-range total cost (776,807 yuan) and the lowest QALYs (4.95 QALYs), as detailed in Table 4.

**Table 4:** Basic results of cost-utility analysis of second-generation AR antagonists in the treatment of mHSPC.

Treatment	Cost (yuan)	Effect (QALYs)	Incremental cost (yuan)	Incremental effect (QALYs)	ICER (yuan/QALY)
Compared with Enza_ADT					
Enza_ADT	872,033	5.10			
Apa_ADT	776,807	4.95	−95,278	−0.15	643,309
Dar_ADT	751,369	5.58	−120,664	0.48	−252,398
Compared with Apa_ADT					
Apa_ADT	776,807	4.95			
Enza_ADT	872,033	5.10	95,278	0.15	643,309
Dar_ADT	751,369	5.58	−25,438	0.63	−40,625

When using 287,391 yuan/QALY (three times the 2024 per capita GDP of China) as the WTP threshold, the results showed that the ICER of darolutamide was −252,398 yuan/QALY compared with enzalutamide and −40,625 yuan/QALY compared with apalutamide. Both ICER values were negative, indicating that the darolutamide regimen can reduce costs while improving health outcomes, making it an absolute dominant strategy in the treatment of mHSPC. On the other hand, compared with apalutamide, the ICER of enzalutamide was as high as 643,309 yuan/QALY. With both the incremental cost and incremental effect being positive, it meant that every additional QALY obtained with enzalutamide required an additional cost of 643,309 yuan, which significantly exceeded the WTP threshold. Therefore, apalutamide exhibited better cost-effectiveness than enzalutamide.

### 3.2 Single-factor sensitivity analysis

**Figure 2:**
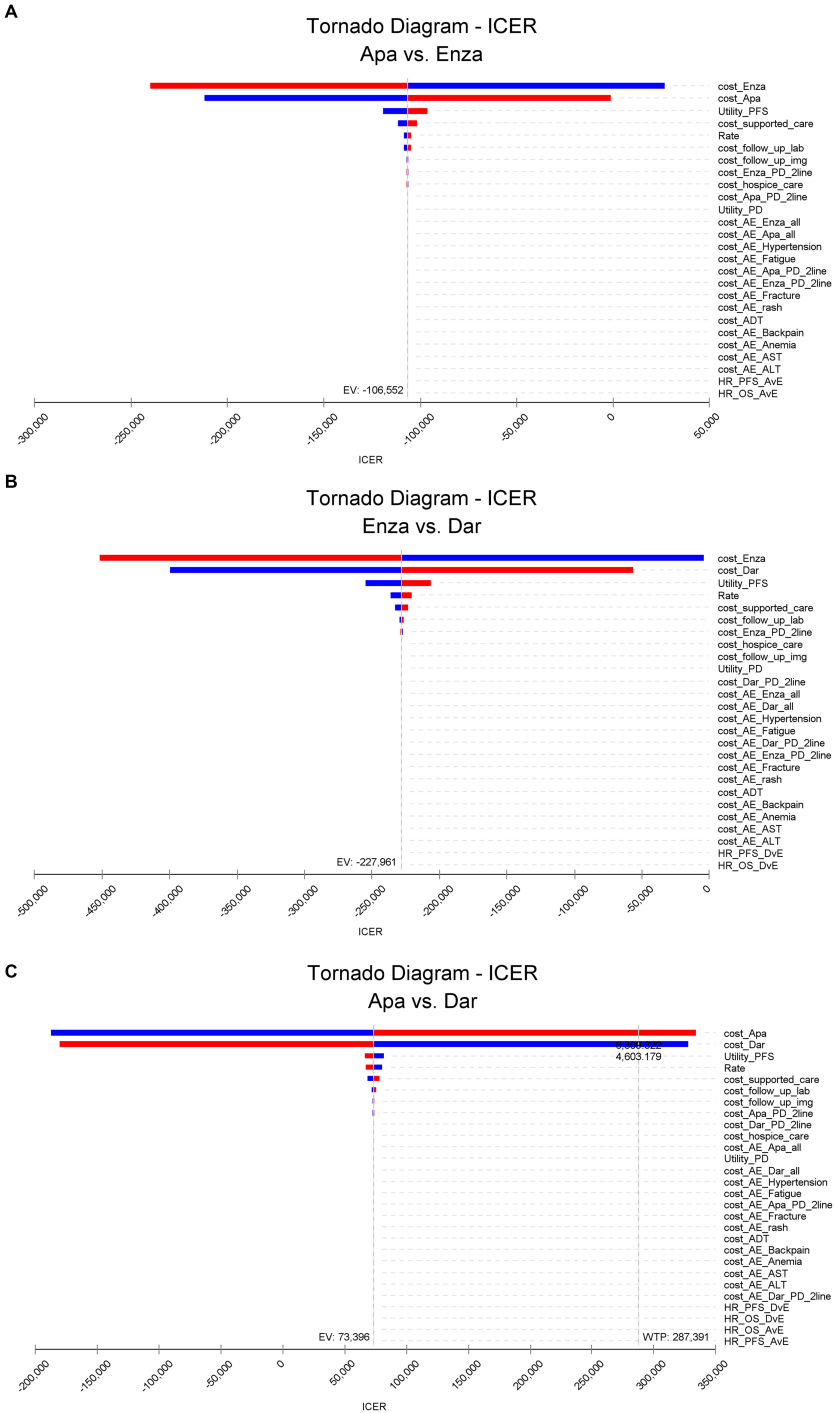
Results of univariate sensitivity analysis.

The results of the univariate sensitivity analysis for pairwise comparisons of apalutamide, darolutamide, and enzalutamide are shown in Figure 2 (include Figures 2A–C). The drug costs for first-line treatment and the health utility value for imaging progression-free survival have a significant impact on the ICER results, followed by the costs of supportive therapies, discount rates, and laboratory biochemical tests during follow-up. The cost of adverse event management and the HR between different treatment regimens have a minor effect on the ICER.

Apalutamide vs. enzalutamide: Apalutamide consistently demonstrates cost-effectiveness superiority across all parameter ranges; darolutamide vs. enzalutamide: Darolutamide’s ICER is consistently below the WTP, showing a greater cost-effectiveness advantage; darolutamide vs. apalutamide: If the drug cost of apalutamide exceeds 6300.322 yuan per cycle, its ICER will surpass the WTP and no longer demonstrate a cost-effectiveness advantage. The univariate sensitivity analysis confirmed the robustness of the base case analysis results.

### 3.3 Probabilistic sensitivity analysis

**Figure 3:**
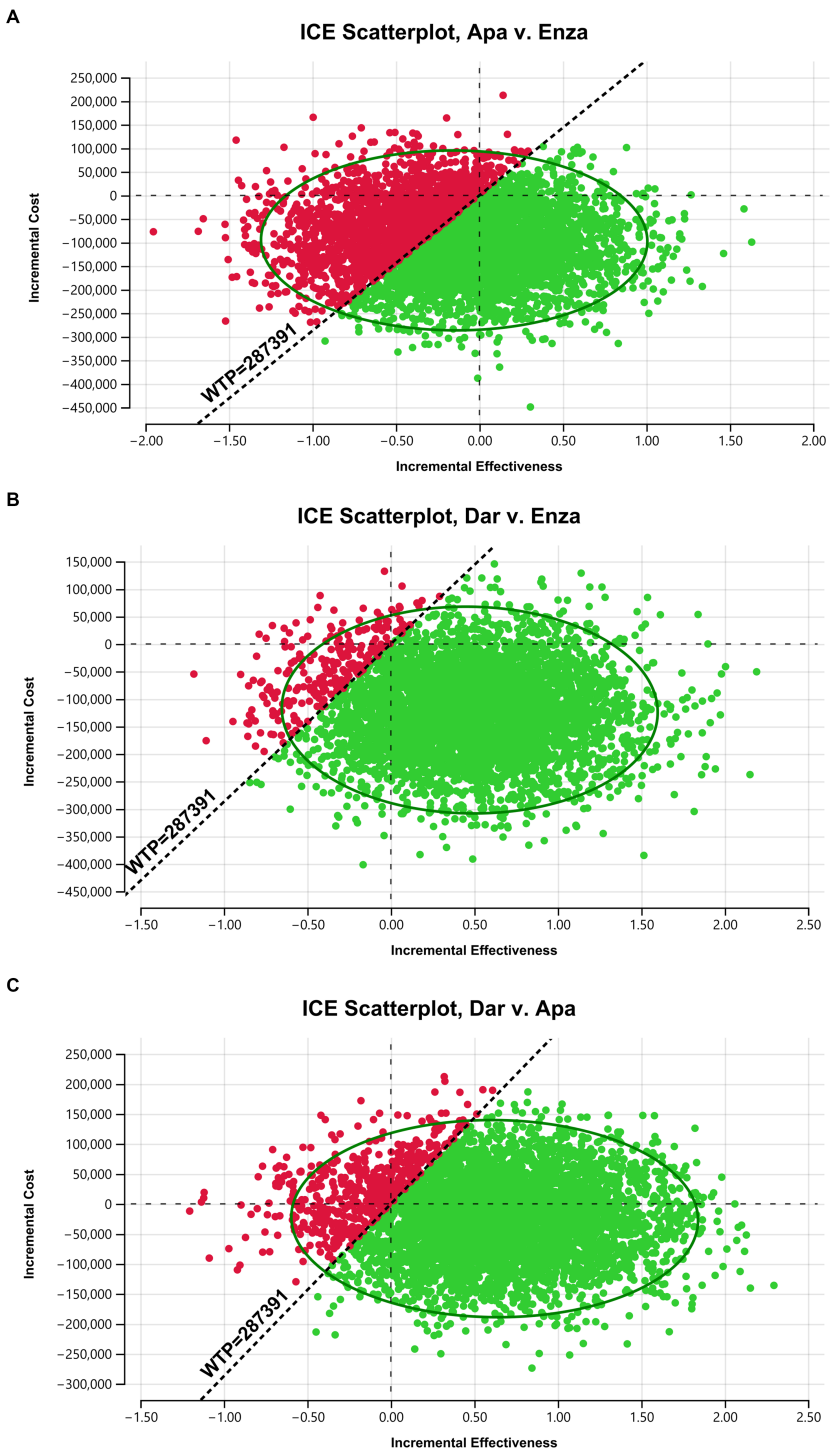
Scatter plot of the cost-effectiveness plane.

The results of the probabilistic sensitivity analysis for pairwise comparisons of apalutamide, darolutamide, and enzalutamide are shown in the scatter plots in Figure 3 (include Figures 3A–C). The *x*-axis represents incremental effectiveness, and the *y*-axis represents incremental cost. The scatter points in each plot represent the ICER values for each sampling of model parameter combinations, which are mostly concentrated within an ellipse, with a small degree of data dispersion. This indicates that the model parameters are robust, and the probabilistic sensitivity analysis results are reliable. When the WTP is 287,391 yuan, compared to enzalutamide, more scatter points for apalutamide or darolutamide fall in the lower-right side below the WTP line, meaning that the probability of apalutamide and darolutamide having a cost-effectiveness advantage in treating mHSPC is higher. In contrast, compared to darolutamide, more scatter points for apalutamide fall above the WTP line, indicating a very low probability of apalutamide having a cost-effectiveness advantage.

**Figure 4:**
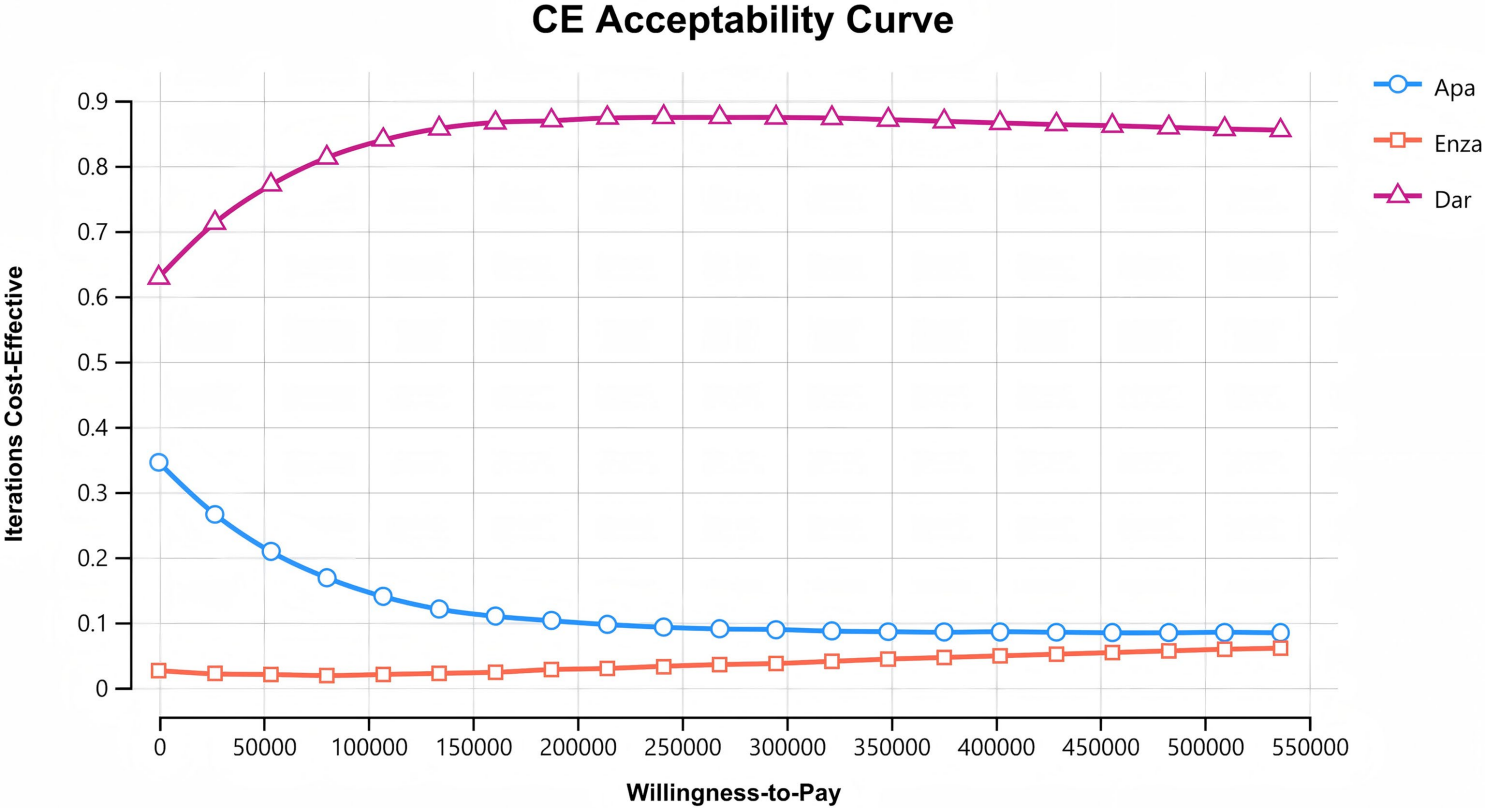
Cost-effectiveness acceptable curve.

After 10,000 random samplings in the Monte Carlo simulation, the cost-effectiveness acceptability curves for the three second-generation AR antagonists are shown in Figure 4. The *x*-axis represents WTP, and the *y*-axis represents the probability of cost-effectiveness. Among the three drugs, darolutamide has the highest likelihood of cost-effectiveness advantage, which increases with WTP and then levels off. The probability of apalutamide having a cost-effectiveness advantage gradually decreases as WTP increases. The likelihood of enzalutamide having a cost-effectiveness advantage increases slowly with WTP, but it is still the lowest of being cost-effective, consistent with the base case analysis results.

## 4 Discussion

The growth of prostate cancer is highly dependent on the androgen signaling pathway, and most patients will eventually relapse and progress to mCRPC (4, 37). Due to the poor prognosis of mCRPC, the quality of life of patients is significantly reduced, and the mortality rate is high. Therefore, delaying the progression of mHSPC to mCRPC is of great importance for improving long-term prognosis and reducing the socio-economic burden.

Second-generation AR antagonists have demonstrated significant efficacy in patients with mHSPC, while their treatment costs remain a critical factor in clinical decision-making and reimbursement considerations. In this study, we evaluated the cost-effectiveness of enzalutamide, apalutamide, and darolutamide in the treatment of mHSPC by constructing a Markov model, providing an evidence-based basis for rational clinical drug use and health policy formulation.

The cost-effectiveness analysis results show that when the WTP threshold is set to three times the per capita GDP of China (287,391 yuan/QALY), the darolutamide regimen has the best cost-effectiveness advantage, and apalutamide offers a better cost-effectiveness advantage compared to enzalutamide. Sensitivity analysis results confirm the robustness of these results. The cost-acceptability curves for all three treatments indicate that when the WTP exceeds 550,000 yuan, the probability of enzalutamide becoming the cost-effective option may be higher than that of apalutamide. Given the outstanding economic advantages of darolutamide combination therapy, it is recommended that, once its indications are approved, darolutamide + ADT be prioritized for inclusion in the national medical insurance catalog and clinical pathway optimization, providing patients with a more cost-effective treatment option. For budget-constrained regions, price negotiations for apalutamide could further reduce costs and enhance accessibility, thereby maximizing public health benefits.

This study also has certain limitations. First, the clinical trial participants included in this study are from multiple regions, with Chinese patients accounting for only a small proportion, and most prostate cancer patients in China are diagnosed at later clinical stages, which introduces some variability. Therefore, it remains unclear whether the efficacy and safety results from these clinical trials can be directly extrapolated to the Chinese population. Second, due to the lack of head-to-head clinical trial data for second-generation AR antagonists in the treatment of mHSPC, this study extrapolated survival curves for the apalutamide and darolutamide groups based on HR obtained from network meta-analysis, which may introduce some uncertainty. Furthermore, in order to simplify the model calculations, the cost of ADT was represented by the cost of leuprolide acetate, and the cost for managing ≥3 AEs was sourced from published literature, which may lead to some bias between the model outputs and real-world data.

In the future, more clinical or real-world studies are still needed, especially localized studies targeting Chinese prostate cancer patients, to further clarify the direct efficacy differences of the second-generation AR antagonists in the treatment of mHSPC and improve the accuracy and applicability of economic assessment.

## 5 Conclusion

When the WTP is ¥287,391, darolutamide combined with ADT is the treatment plan with the most cost-effectiveness advantage for the treatment of mHSPC. It not only delays disease progression and improves the quality of life of patients but also significantly saves medical resources.

## Data Availability

The original contributions presented in the study are included in the article/supplementary material, further inquiries can be directed to the corresponding author.
